# Development and Growth of the Avian Pectoralis Major (Breast) Muscle: Function of Syndecan-4 and Glypican-1 in Adult Myoblast Proliferation and Differentiation

**DOI:** 10.3389/fphys.2017.00577

**Published:** 2017-08-08

**Authors:** Sandra G. Velleman, Yan Song

**Affiliations:** ^1^Department of Animal Sciences, The Ohio State University Wooster, OH, United States; ^2^Department of Medical Oncology, Dana-Farber Cancer Institute, Harvard Medical School Boston, MA, United States

**Keywords:** glypican-1, microRNA, muscle, satellite cell, syndecan-4

## Abstract

Muscle fiber number is determined around the time hatch with continued posthatch muscle growth being mediated by the adult myoblast, satellite cell, population of cells. Satellite cells are dynamic in their expression of proteins including the cell membrane associated proteoglycans, syndecan-4 and glypican-1. These proteoglycans play roles in organizing the extracellular environment in the satellite cell niche, cytoskeletal structure, cell-to-cell adhesion, satellite cell migration, and signal transduction. This review article focuses on syndecan-4 and glypican-1 as both are capable of regulating satellite cell responsiveness to fibroblast growth factor 2. Fibroblast growth factor 2 is a potent stimulator of muscle cell proliferation and a strong inhibitor of differentiation. Proteoglycans are composed of a central core protein defined functional domains, and covalently attached glycosaminoglycans and N-glycosylation chains. The functional association of these components with satellite cell function is discussed as well as an emerging role for microRNA regulation of syndecan-4 and glypican-1.

## Introduction

The extracellular matrix is an organized structure that can be either located outside cells or directly associated with the cell membrane. Collagens, proteoglycans, and non-collagenous glycoproteins compose the extracellular matrix. The protein constituents of the extracellular matrix are dynamically expressed and are directly associated with cell proliferation, adhesion, migration, and regulation of cell shape. The extracellular matrix produced by a tissue changes with age, injury, and is tissue-specific. “Traditionally, the extracellular matrix was described as a ground substance that the cells were embedded in and functioned as a structural framework for the cells but did not biologically influence cell behavior (Velleman, 2012).” Research beginning in the 1980s with the advent of molecular tools has shown that the extracellular matrix is critically important in the physiological function of most tissues by forming a signaling loop with the cell responding to extracellular matrix signals with altered gene expression, changes in cell shape, or adhesive properties. Hence, the extracellular matrix produced by the cells within a tissue is creating an extrinsic environment that governs its own behavior.

Communication between the extracellular matrix and muscle cells plays a pivotal role in the regulation of muscle cell proliferation and differentiation. Proliferation represents the replication of muscle cells available to fuse and differentiate into multinucleated myotubes whereas differentiation refers to the formation of muscle specific structures including multinucleated myotubes and fibers. Increased proliferation will provide a larger pool of muscle cells available for differentiation. Changes in the level of differentiation will affect muscle fiber size and the number of myofibers.

In addition to the muscle fibers, there are three layers of connective tissue in mature skeletal muscle: the endomysium, perimysium, and epimysium. The endomysium separates individual muscle fibers, the perimysium surrounds bundles of muscle fibers, and the epimysium forms a sheath around the entire muscle. These connective tissue layers are composed of cells and extracellular matrix proteins. The predominant extracellular matrix protein in the connective tissue layers are collagens. Types I, III, IV, V, and VI collagen are found in skeletal muscle (Nishimura et al., 1997). Although the collagens play important structural and functional roles in extracellular matrix regulation of muscle growth properties, this review will focus on the cell membrane associated proteoglycans, syndecan-4 and glypican-1, due to their function in regulating the muscle cell growth processes including muscle cell proliferation and differentiation.

## What are proteoglycans?

“Proteoglycans are a varied group of proteins containing a central core protein and at least one covalently-attached glycosaminoglycan (GAG) chain. The central core protein ranges in size from ~40,000 to >350,000 daltons (Iozzo and Murdoch, 1996; Iozzo, 1998; Velleman, 2012).” The definition of a proteoglycan is very broad and will include macromolecules with a variety of biological roles including tissue hydration, organization of the tissue architecture, regulation of gene expression, cell proliferation and differentiation, cell migration, cell adhesion, and transduction of extracellular signals to the cell. The impact of proteoglycans on cellular behavior is immense with all of these processes being essential for muscle development and growth. Proteoglycan biological activity is not limited to just the central core protein but involves the post-translational addition of the GAGs and N-glycosylation chains.

“The GAG chains are polymers of disaccharide repeats that are sulfated and contain a high negative charge. The negative charge permits ionic interactions with molecules such as water or growth factors. Glycosaminoglycan chains attached to the core protein include chondroitin sulfate, dermatan sulfate, keratan sulfate, and heparan sulfate (Velleman, 2012).” Glycosaminoglycans preferentially attach to repeat amino acid sequences Serine-Glycine-Serine or Serine-Glycine-Serine-Glycine which is the primary acceptor site for xylosyltransferase to initiate the addition of GAGs to the core protein (Bourdon et al., 1987; Zhang et al., 1995). “Chondroitin sulfate is composed of repeats of glucuronic acid and N-acetylglucosamine with sulfate groups in the 4- and 6- position of the amino sugar. Heparan sulfate consists of repeats of glucuronic acid and N-acetylglucosamine. Keratan sulfate contains disaccharide repeats of galactose and N-acetylglucosamine with the sulfate at the 6-position of the amino sugar (Velleman, 2012).” The high negative charge of the GAGs allows them to ionically interact with many molecules including but not limited to water and growth factors. The N-glycosylation chains are attached to the core protein at an asparagine amino acid at the sequence Asparagine-Xaa-Serine/Threonine. The Xaa can be any amino acid except proline (Kornfeld and Kornfeld, 1985). N-glycosylation chains are involved in proper folding of proteins (Parodi, 2000) and localization of membrane proteins to the cell surface (Martinez-Maza et al., 2001). In skeletal muscle, proteoglycan expression changes during muscle development and growth from one rich in large chondroitin sulfate proteoglycans to a mixture of chondroitin, dermatan, and heparan sulfate proteoglycans immediately preceding hatch (Young et al., 1989; Fernandez et al., 1991; Velleman et al., 1999). This expression pattern of proteoglycans indicates that different proteoglycans may have distinct developmental functions during the muscle growth process.

## Heparan sulfate proteoglycans: the syndecans and glypicans

Two major groups of membrane-associated heparan sulfate proteoglycans, the syndecans and glypicans, are found in skeletal muscle. To date there are four members of the syndecan family, 1–4 (Larrain et al., 1997; Brandan and Larrain, 1998; Fuentealba et al., 1999; Liu et al., 2006). The 4 syndecans share common structural features. The syndecans all have an N-terminal signal peptide, an extracellular domain that contains attachment sites for the GAGs, a transmembrane domain, and a C-terminal cytoplasmic domain containing conserved domain 1, variable (V) domain, and conserved domain 2. The transmembrane and cytoplasmic domains are conserved across species and within the syndecan family. The ectodomain containing the GAG binding sites and N-glycosylation sites is less conserved. The syndecans are primary modulators of cellular behavior associated with both cell proliferation and differentiation (Velleman and Liu, 2006).

Syndecan-4 is the most widely distributed of the syndecans (Couchman, 2010). Although similar in structure to syndecans-1 through -3, syndecan-4 has several unique features. For example, the extracellular domain of syndecan-4 can be shed from the cell surface, and the cytoplasmic domain can bind to protein kinase C alpha (PKCα) and phosphatidylinositol 4,5-biphosphate (PIP_2_) (Oh et al., 1997a,b, 1998). Protein kinase C alpha binds to the V region of the syndecan-4 cytoplasmic domain through the intermediate PIP_2_ (Oh et al., 1997a,b, 1998; Horowitz et al., 1999). In muscle, syndecan-4 has been shown to play a key role in mechanisms modulating skeletal muscle development and will be the focus of the following discussion.

The glypicans, unlike the syndecans, are not transmembrane heparan sulfate proteoglycans, but attached to the cell surface through a glycosylphosphoinositol (GPI) anchor. There are six vertebrate glypicans: glypican-1 through -6. The glypicans contain an N-terminal signal sequence followed by a globular domain containing multiple cysteine residues, a GAG binding domain, N-glycosylation sites, and a C-terminus GPI anchor domain leading to the attachment of glypican to the cell surface. Unlike the syndecans which can make direct contact with internal cytoskeletal components through their cytoplasmic domain to activate signal transduction pathways, glypican activation of signaling pathways is not indirect and involves other transmembrane molecules, since glypican does not contain a transmembrane domain (Velleman and Liu, 2006). Only glypican-1 has been shown to play a role in myogenesis through its regulation of fibroblast growth factor 2 (FGF2).

## Function of syndecan-4 and glypican-1 attached heparan sulfate chains and N-glycosylation chains in myogenesis

The covalently attached heparan sulfate chains to the syndecan-4 and glypican-1 core proteins are involved in the biological activity of these proteoglycans. For example, growth factors are strong stimulators or inhibitors of myoblast and myogenic satellite cell proliferation and differentiation. Fibroblast growth factor 2 is a potent stimulator of muscle cell proliferation and a strong inhibitor of differentiation into muscle specific structures (Dollenmeier et al., 1981). Myogenin, a muscle specific transcriptional regulatory factor, required for the initiation of myotube formation (Brunetti and Goldfine, 1990) is inhibited by FGF2. By suppressing myogenin expression, FGF2 maintains the skeletal muscle cells in a state of proliferation increasing the available pool of muscle cells for muscle fiber formation. For FGF2 to interact with its tyrosine kinase receptor, it must bind to heparan sulfate chains attached to proteoglycans like syndecan or glypican. If the heparan sulfate chains are removed from the proteoglycan core protein, FGF2 will no longer inhibit muscle differentiation (Rapraeger et al., 1991).

**Figure 1:**
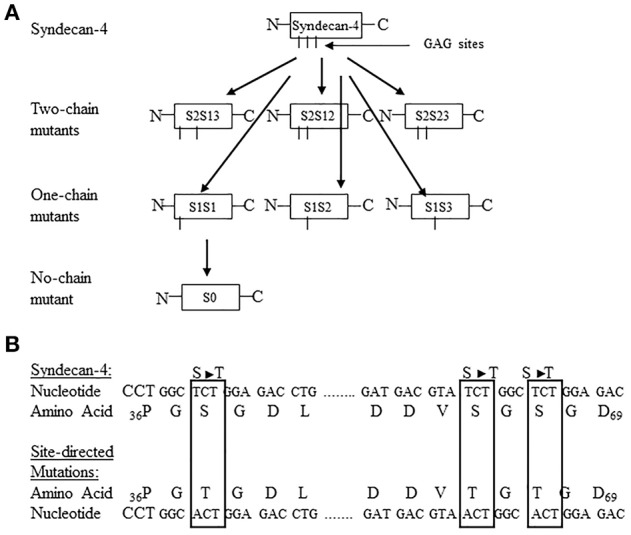
**(A)** Site-directed mutagenesis strategy used for turkey syndecan-4. The 3 potential glycosaminoglycan (GAG) attachment sites are located at Ser_38_, Ser_65_, and Ser_67_ and are referred to as chain 1, chain 2, and chain 3, respectively. The following nomenclature was developed to identify the specific site-directed mutants: S = syndecan-4, the number after S refers to the number of potential GAG sites unaltered, and the number after hyphen indicates the GAG attachment sites not modified. For example, S2–13 means two-chain of syndecan-4 at the Ser_38_, and _67_ sites intact. By the same token, S1-1 would refer to a syndecan-4 with one-chain intact at the Ser_38_, and S0 represents the zero chain presented in the construct. All other abbreviations follow this format. The schematic shows the generation of all possible two-chain mutants, one-chain mutants, and no-chain mutant. **(B)** Partial nucleotide and amino acid sequence of turkey syndecan-4 including the potential GAG attachment sites showing the conversion of Ser (S) to Threonine (T). The nucleotide and resulting amino acid sequence s for the site-directed mutated sites are highlighted in the boxes (Figure reproduced from Zhang et al., 2008).

**Figure 2:**
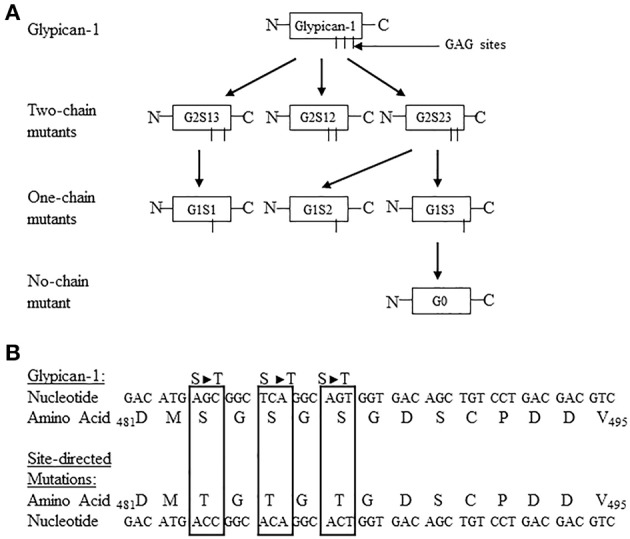
**(A)** Site directed mutagenesis strategy used for turkey glypican-1. The three potential glycosaminoglycan (GAG) attachment sites are located at Ser_483_, Ser_485_, and Ser_487_. The Ser_483_ is referred to as chain 1, Ser_485_ is chain 2, and Ser_487_ is chain 3. The following nomenclature was developed to identify the specific site directed mutants: G = glypican-1, the number after G refers to the number of potential GAG sites unaltered, S = serine, and the number after S indicates the GAG attachment sites not modified. For example, G2S12 means Glypican-1 with the Ser_483_ and _485_ sites not mutated, and G1S2 would refer to a glypican-1 one-chain mutant containing the Ser_485_ site, and G0 represents the no-chain mutant construct. All other abbreviations follow this rule. The schematic shows the generation of all possible two-chain mutants, one-chain mutants, and no-chain mutant. **(B)** Partial nucleotide and amino acid sequence of turkey glypican-1 including the potential GAG attachment sites at Ser_483_, Ser_485_, and Ser_487_. The nucleotide and resulting amino acid sequences for the site-directed mutated sites are highlighted in the boxes. (Figure reproduced from Zhang et al., 2007).

The function of the individual heparan sulfate chains attached to syndecan-4 and glypican-1 core proteins has remained an enigma. To further define the functional contribution of the covalently attached heparan sulfate chains to the syndecan-4 and glypican-1 core proteins, a site directed mutagenesis approach was developed targeting each of the syndecan-4 and glypican-1 heparan sulfate chains (Zhang et al., 2007, 2008). Syndecan-4 has 3 heparan sulfate attachment sites in its core protein at serine residues 38, 65, and 67 (Zhang et al., 2008). Glypican-1 has 3 heparan sulfate attachment sites at serine residues 483, 485, and 467 (Zhang et al., 2007). For both syndecan-4 and glypican-1, site directed mutant constructions were created leaving only 1 heparan sulfate chain attached to each of the serine residues or no heparan sulfate chains attached (Zhang et al., 2007, 2008). Figures 1, 2 are schematic representations of the site directed mutagenesis cloning strategy used for syndecan-4 and glypican-1.

The activity of each of the GAG attachment sites were studied by Velleman et al. (2007) and Zhang et al. (2008) during proliferation and differentiation, and the responsiveness to FGF2 in turkey breast muscle satellite cells. The satellite cells were transfected with expression vector constructs of wild-type syndecan-4, one-chain mutant, no-chain mutant or the empty vector. All of the constructs inhibited cell proliferation and delayed initial differentiation but did not affect the responsiveness of the cells to FGF2 treatments. Taken together, these data showed that syndecan-4 in turkey breast muscle satellite cells can function in an FGF2-independent manner and its function is not completely derived from the covalently attached heparan sulfate chains.

Similar experiments were conducted by Zhang et al. (2007) on glypican-1 function during proliferation and differentiation, and responsiveness to FGF2. Unlike syndecan-4 the overexpression of wild-type glypican-1 increased responsiveness to FGF2 during proliferation compared to the one-chain and no-chain mutants, but did not affect proliferation compared to the empty-vector control. Glypican-1, in breast muscle satellite cells, functions in an FGF2-dependent manner, whereas syndecan-4 can elicit a biological affect in an FGF2-independent manner. Furthermore, when glypican-1 is released from the cell surface by cleavage of its GPI anchor it can still bind FGF2 to its heparan sulfate chains (Velleman et al., 2013). When FGF2 is sequestered away from the cell surface, it is no longer able to bind to its tyrosine kinase receptor and activate cellular signal transduction pathways.

Historically, proteoglycan research has primarily addressed the biological impact of the attached GAG chains, due to their high negative charge and resulting ionic interactions with both molecules like water and growth factors, and more recently the proteoglycan central core protein. Many proteoglycans contain N-glycosylation chains attached to the core protein, but the function of the N-glycosylation chains has largely been overlooked as they are a minor component of the entire proteoglycan structure. N-glycosylation chains have an important biological function in protein activity. Properties attributed to N-glycosylation chains include protein folding (Parodi, 2000), and localization of cell surface proteins (Martinez-Maza et al., 2001).

Syndecan-4 has 2 N-glycosylation chains attached to the asparagine (Asn) residue of amino acid sequence Asn-Xaa-Serine/Threonine; Xaa can be any amino acid except proline (Kornfeld and Kornfeld, 1985) at Asn residues 124 and 139. The overexpression of syndecan-4 glycosylation mutants with or without the heparan sulfate chains did not change satellite cell proliferation, differentiation, or the ability to respond to FGF2 compared to wild-type syndecan-4 (Song et al., 2011). However, during proliferation overexpression of the N-glycosylation chain mutants without the heparan sulfate chains increased myogenic satellite cell proliferation suggesting that syndecan-4 plays a more prominent role during proliferation and both the heparan sulfate and N-glycosylation chains are important in muscle development mediated by syndecan-4.

**Figure 3:**
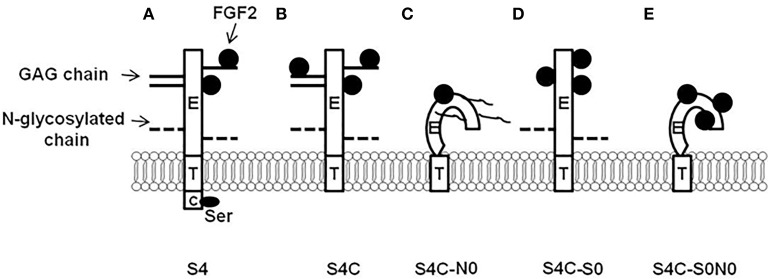
Syndecan-4 cytoplasmic domain regulation of cellular responsiveness to fibroblast growth factor 2 (FGF2). **(A)** Syndecan-4 can bind FGF2 to its core protein and GAG chains, and then present FGF2 to its receptor to initiate cellular responsiveness to FGF2; **(B)** The deletion of syndecan-4 cytoplasmic domain may cause increased binding of FGF2 to syndecan-4 (Ser residue in the conserved 1 region is critical in this process). **(C)** The deletion of syndecan-4 N-linked glycosylated (N-glycosylation) chains changes the three dimensional structure of the core protein which may lead to the decreased interaction with FGF2; **(D)** The deletion of syndecan-4 glycosaminoglycan (GAG) chains gives more spaces for the core protein to bind to FGF2; and **(E)** The deletion of both syndecan-4 N-glycosylation chains and GAG chains changes the three dimensional structure of the core protein which deceases FGF2 binding to syndecan-4, however, the deletion of the GAG chains give more space for the core protein to bind to FGF2 which rescues the interaction between syndecan-4 and FGF2. S4, wild type syndecan-4; S4C, syndecan-4 without cytoplasmic domain; S4C-N0, syndecan-4 without cytoplasmic domain and N-glycosylation chains; S4C-S0, syndecan-4 without cytoplasmic domain and GAG chains; and S4C-S0N0, syndecan-4 without cytoplasmic domain, N-glycosylation chains, and GAG chains. (Figure reproduced from Song et al., 2012b).

In addition, to the biological function of syndecan-4 requiring the heparan sulfate and N-glycosylation chains, the syndecan-4 core protein cytoplasmic domain also regulates syndecan-4 signal transduction. Song et al. (2012b) studied how the syndecan-4 cytoplasmic domain in combination with the heparan sulfate chains and N-glycosylation chains regulates the proliferation, differentiation, and FGF2 responsiveness of satellite cells. The results from this study demonstrated that the syndecan-4 cytoplasmic domain, heparan sulfate chains, and N-glycosylation chains were important in the proliferation and not the differentiation of the myogenic satellite cells by modulating FGF2 responsiveness, and the localization of PKCα. The following possible cellular mechanisms from the various combinations of heparan sulfate, N-glycosylation chains, and cytoplasmic domain mutants have been proposed for syndecan-4 (Figure 3). In Figure 3A, syndecan-4 binds FGF2 to its heparan sulfate chains and core protein with the cytoplasmic domain interacting with PIP_2_. Deletion of the syndecan-4 cytoplasmic domain depicted in Figure 3B may increase the binding of FGF2 to the heparan sulfate chains and core protein as the serine residue in the C1 region of the cytoplasmic domain is phosphorylated by FGF2 (Horowitz and Simons, 1998a). Removal of the N-glycosylation chains as shown in Figure 3C may affect the secondary structure of syndecan-4 as N-glycosylation chains are involved in the proper folding of proteins (Parodi, 2000) and membrane localization (Martinez-Maza et al., 2001). The deletion of the heparan sulfate chains in Figure 3D will just leave the core protein as a site for the binding of FGF2. Figure 3E depicts the syndecan-4 core protein without the heparan sulfate and N-glycosylation chains. Thus, core protein three dimensional structure will be altered and FGF2 will only be able to bind to the core protein. In Figures 3B–E, the cytoplasmic domain is deleted from the syndecan-4 core protein. Deletion of the cytoplasmic domain will also affect the biological function of syndecan-4 by impacting cellular signal transduction.

Glypican-1 has 3 N-linked glycosylated chains attached to the core protein at Asn amino acid residues 76, 113, and 382 (Song et al., 2010). Glypican-1 site directed mutants were created containing one of each of the N-glycosylation chains, a mutant without any N-glycosylation chains, and N-glycosylation chain 1-chain and no-chain mutants with or without attached core protein heparan sulfate chains. The glypican-1 N-glycosylation 1-chain and no-chain mutants without the heparan sulfate chains were transfected into turkey myogenic satellite cells to examine the interaction of glypican-1 N-glycosylation chains and the heparan sulfate chains in the proliferation, differentiation, and responsiveness to FGF2 (Song et al., 2010). The overexpression of glypican-1 N-glycosylation 1-chain and no-chain mutants without the heparan sulfate chains increased the proliferation and differentiation of the satellite cells compared to the wild-type glypican-1. However, a similar affect was not observed with the glypican-1 N-glycosylation 1 chain constructs with the heparan sulfate chains attached to the core protein. Interestingly, overexpressing the glypican-1 N-glycosylation chain constructs with or without the heparan sulfate chains increased satellite cell responsiveness to FGF2 compared to wild-type glypican-1. Thus, the N-glycosylations are likely involved in glypican-1 FGF2 responsiveness.

**Figure 4:**
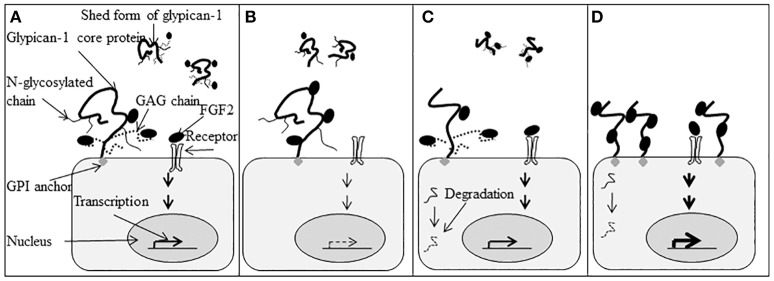
Function of glypican-1 N-linked glycosylated (N-glycosylation) chains and glycosaminoglycan (GAG) chains in fibroblast growth factor 2 (FGF2) signaling. **(A)** Glypican-1 has both membrane-associated and shed forms. The membrane-associated form is attached to the cell membrane by a glycosylphosphatidylinositol (GPI) anchor. Glypican-1 has three N-glycosylation chains and three GAG chains. The N-glycosylation chains are attached to the core protein at Asn^76^, Asn^113^, and Asn^382^. The Asn^76^ is referred to as the N1 chain, Asn^113^ is the N2 chain, and Asn^382^ is the N3 chain. Both glypican-1 core protein and GAG chains can bind and present FGF2 to its tyrosine kinase receptor and initiate cell signaling into the nuclei to stimulate gene transcription that influences cell proliferation and differentiation. **(B)** When GAG chains are deleted, glypican-1 core protein can still bind and present FGF2 to its tyrosine kinase receptor and initiate cell signaling. The cell has lower responsiveness to FGF2. **(C)** The deletion of glypican-1 N-glycosylation chains will change the three-dimensional structure of glypican-1 core protein which may influence glypican-1 localization on the cell membrane. Fibroblast growth factor 2 signaling will be initiated normally in the presence of the glypican-1 core protein and GAG chains. The protein without folding properly may be degraded (
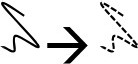
). **(D)** Glypican-1 N-glycosylation mutants without GAG chains cannot be released from the cell membrane normally. The increased number of glypican-1 core proteins bound to the cell surface will increase cell responsiveness to FGF2. Transcription levels are indicated by the density of the arrows (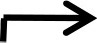
indicates normal transcription level, 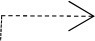
indicates reduced transcription level, and 
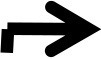
indicates increased transcription level). (Figure reproduced from Song et al., 2010).

Figure 4 is a schematic illustration highlighting the function of the glypican-1 N-glycosylation and heparan sulfate chains in the biological function of glypican-1. Figure 4A depicts expected glypican-1 signal transduction. In Figure 4B, the heparan sulfate chains are deleted but the core protein can still bind FGF2 and present it to its tyrosine kinase receptor with reduced responsiveness to FGF2. Removal of the N-glycosylation chains in Figure 4C will result in changes to the three dimensional structure of the glypican-1 core protein. Without appropriate secondary and tertiary structure, the protein may be degraded or be altered in its cell surface localization. As depicted in Figure 4D, glypican-1 without the heparan sulfate or N-glycosylation chains will likely not be shed from the cell surface but can still present FGF2 to its tyrosine kinase receptor as FGF2 can still bind directly to the glypican-1 core protein.

## Syndecan-4 function in focal adhesion formation

Focal adhesions are areas where muscle cells are observed to be tightly attached to the extracellular matrix. The focal adhesions are composed of many proteins including but not limited to: (i) extracellular matrix molecules that cells attach to; (ii) transmembrane receptors including integrins (Hynes, 1992; Schwartz et al., 1995), cadherins (Takeichi, 1988, 1990, 1991), selectins (Bevilacqua and Nelson, 1993) and syndecan-4; (iii) cytoplasmic structural proteins such as β-actin, talin, vinculin, α-actinin, paxillin, and tensin; and (iv) the signaling proteins including focal adhesion kinase (FAK), PKCα (Clark and Brugge, 1995; Burridge and Chrzanowska-Wodnicka, 1996) and Src (Clark and Brugge, 1995; Burridge and Chrzanowska-Wodnicka, 1996). Focal adhesions act not only as anchorage points for the cells, but also mediate mechanical and biochemical signaling.

Integrins are the primary factors for assembling focal adhesions. In muscle cells, integrins are involved in cell proliferation, adhesion, and apoptosis (Liu et al., 2008). In the process of focal adhesion formation, heparan sulfate proteoglycans are also required (Woods et al., 1986). For example, syndecan-4 was reported to play an important role in promoting focal adhesion formation (Woods and Couchman, 1994; Echtermeyer et al., 1999). Saoncella et al. (1999) described that syndecan-4 is critical for integrin mediated fibronectin induced focal adhesion formation and stress fiber assembly. Syndecan-4 and integrins cooperate with each other to regulate focal adhesion formation and modulate cell migration (Couchman, 2003; Morgan et al., 2007). The interaction between integrins and syndecans is comprehensively reviewed by Roper et al. (2012). In muscle stem cells, overexpression of syndecan-4 decreased cell proliferation (Zhang et al., 2008; Song et al., 2011, 2012b). It is possible that more focal adhesions were formed and cell migration was suppressed with the overexpression of syndecan-4. Because migration is essential for muscle stem cells to interact with each other and exchange signals for proliferation, the increased number of focal adhesions inhibited cell proliferation. This is evidenced by Longley et al. (1999) who used Chinese hamster ovary K1 cells which showed greater expression of syndecan-4 increased focal adhesion formation and decreased cell migration.

The syndecan-4 core protein, covalently attached heparan sulfate chains, and the N-linked glycosylated chains are all involved in focal adhesion formation (Song et al., 2012c). The cytoplasmic domain of syndecan-4 is critical in focal adhesion formation and signal transduction (Echtermeyer et al., 1999; Saoncella et al., 1999; Woods et al., 2000; Woods and Couchman, 2001; Song et al., 2012d). Overexpression of syndecan-4 induced more focal adhesion formation, whereas the overexpression of a mutant syndecan-4 without the V region of the cytoplasmic domain decreased focal adhesion formation (Longley et al., 1999). Syndecan-4 heparan sulfate chains have been reported to bind to the heparin-binding domain of fibronectin in intergrin-mediated focal adhesion formation (Lyon et al., 2000; Woods et al., 2000). Furthermore, Woods et al. (2000) and Woods and Couchman (2001) demonstrated that syndecan-4 heparan sulfate chains function in the initial binding steps of syndecan-4 to the focal adhesion complex. This is supported by the results of LeBaron et al. (1988) that demonstrated GAG-deficient syndecan-4 reduces actin stress fiber assembly and focal adhesion formation. The important role of syndecan-4 N-glycosylation chains in focal adhesion formation was demonstrated by comparing overexpression of wild-type syndecan-4 to syndecan-4 deficient N-glycosylation chains in turkey breast muscle satellite cells (Song et al., 2012c). Overexpression of wild-type syndecan-4 increased FAK activity and deletion of the N-glycosylation chains decreased this effect.

Mechanistic studies have shown that syndecan-4 may regulate focal adhesion formation through enhancing FAK activity (Guan et al., 1991; Kornberg et al., 1992) or function by activating PKCα (Oh et al., 1997a; Lim et al., 2003; Keum et al., 2004). Focal adhesion kinase is a non-receptor tyrosine kinase which functions as a scaffold for focal adhesion components such as Src, Cas, and paxillin (Hanks et al., 1992; Schaller et al., 1992, 1999; Xing et al., 1994; Polte and Hanks, 1995). Its activity can be regulated by syndecan-4 (Wilcox-Adelman et al., 2002). Altered activity of FAK changes the number and size of the focal adhesions (Pirone et al., 2006; Israeli et al., 2010), which can influence cell migration, proliferation, differentiation, survival, and cell signaling pathways (Giancotti and Ruoslahti, 1999; Jeong et al., 2001; Wozniak et al., 2004; Goffin et al., 2006).

Protein kinase C alpha has been reported to affect the formation of focal adhesions (Haller et al., 1998) whose activity can be regulated by the serine residue in the C1 region of the syndecan-4 cytoplasmic domain. The dephosphorylation of the serine residue increases the binding affinity of PIP2 to the V region of syndecan-4, and increases PKCα activity (Couchman et al., 2002; Murakami et al., 2002). The tyrosine residue (amino acid sequence KKPIYKK) in the V region of the syndecan-4 cytoplasmic domain mediates PKCα activity by binding PIP2 (Oh et al., 1998; Horowitz et al., 1999; Couchman et al., 2002) that robustly activates PKCα (Oh et al., 1997b, 1998; Horowitz and Simons, 1998a; Couchman et al., 2002), and alters stress fiber organization including focal adhesion formation (Sun and Rotenberg, 1999). Song et al. (2012a) reported that either deletion of the whole syndecan-4 cytoplasmic domain or the mutation of Ser/Tyr residues resulted in altered PKCα activity.

## Signal transduction pathways

As a transmembrane receptor, syndecan-4 participates in signal transduction in two ways: (i) by transmitting signals from the extracellular matrix into the cell and; (ii) by binding and activating intracellular proteins and related downstream signals including Rho family of GTPases, mTOR (mammalian target of rapamycin)-AKT1 (Protein Kinase B), and Wnt.

**Figure 5:**
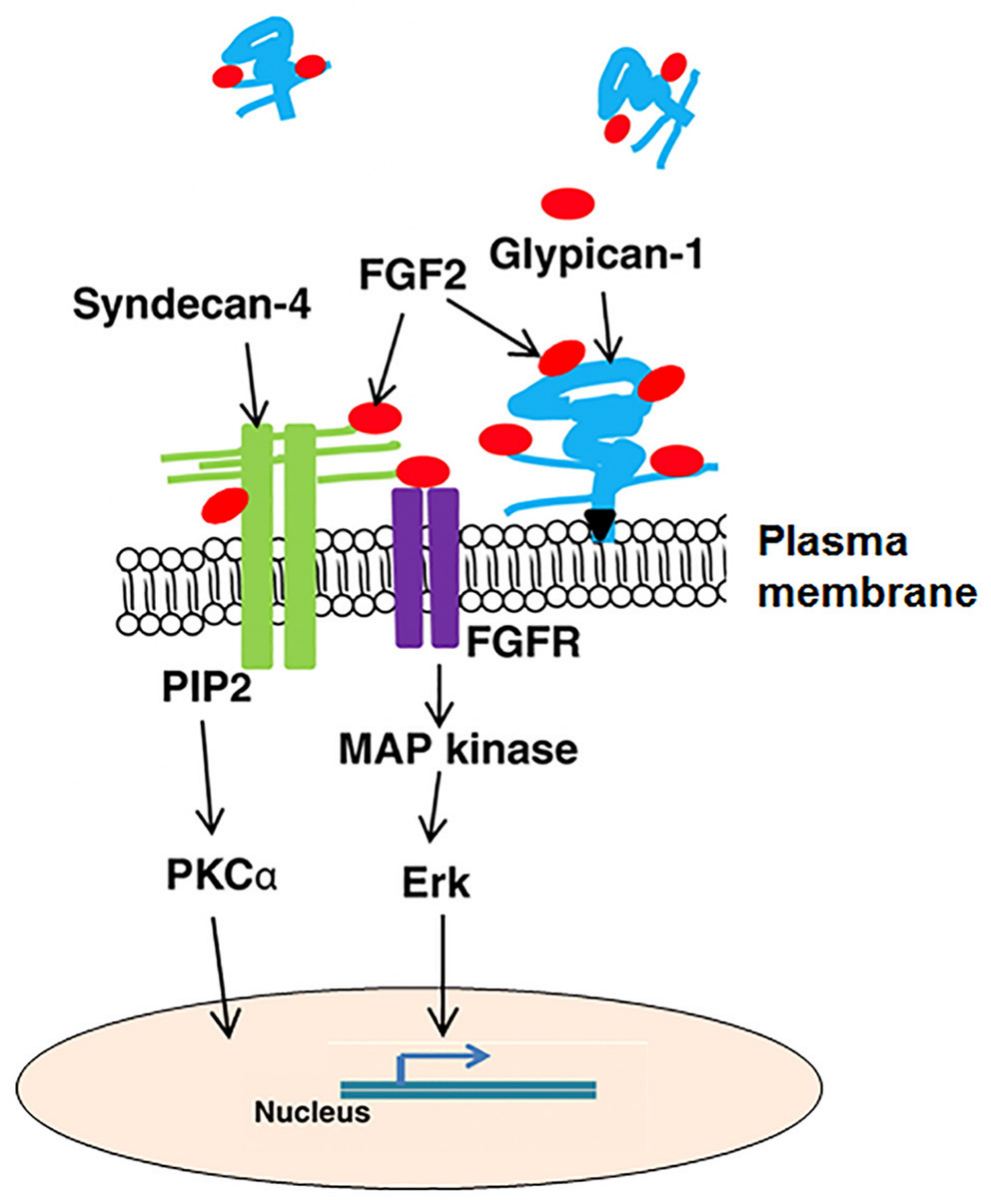
A generalized illustration of pathways, growth factors, and extracellular matrix interactions affected by syndecan-4 and glypican-1. FGF2, fibroblast growth factor 2; FGFR, fibroblast growth factor receptor; MAP kinase, mitogen-activated protein kinase; PI3 kinase, phosphatidylinositol 3-kinase; and PKCα, protein kinase C α.

Syndecan-4 can interact with heparan-binding growth factors including FGF2, hepatocyte growth factor (HGF), vascular endothelial growth factor (VEGF), and platelet-derived growth factors (PDGFs). The lipid raft localization of syndecan-4 oligomers initiates numerous downstream signaling events (Tkachenko and Simons, 2002). By binding FGF2 to its heparan sulfate chains, syndecan-4 concentrates FGF2 on the cell surface and then presents FGF2 to FGF tyrosine kinase receptors (FGFR; Steinfeld et al., 1996) and stabilizes their interaction. The binding of FGF2 phosphorylates FGFR, and then activates Raf-1 and mitogen-activated protein kinase which modulates gene transcription in the nucleus (Figure 5). This aspect is similar in other FGFs as well as HGF, VEGF, and PDGFs.

Syndecan-4 translocates PKCα to the cell membrane and activates it with the assistance of PIP2 (Oh et al., 1998). The binding of PIP2 to the V region of syndecan-4 cytoplasmic domain facilitates the oligomerization of syndecan-4 (Oh et al., 1997a, 1998; Lee et al., 1998; Shin et al., 2012). Syndecan-4 oligomers, not the monomer, promotes the binding of PKCα catalytic domain to the V region of syndecan-4 cytoplasmic domain which activates PKCα (Oh et al., 1997a,b; Horowitz and Simons, 1998a,b). Once activated, PKCα initiates downstream signaling to activate RhoA and Rho kinase to regulate stress fiber formation and maintenance (Woods et al., 2000; Dovas et al., 2006).

The cytoplasmic domain of syndecan-4 is critical in regulating PKCα activity. Song et al. (2012b) demonstrated that the deletion of the syndecan-4 cytoplasmic domain decreased PKCα activity. Furthermore, the serine in the cytoplasmic domain is critical in regulating PIP2 binding and oligomerization status of syndecan-4 (Song et al., 2012a). The serine can be dephosphorylated in the presence of FGF2, then promote syndecan-4 oligomerization, and enhance PKCα activity and downstream signaling pathways (Horowitz and Simons, 1998a; Rybin et al., 1999). The phosphorylation status of the serine can also be mediated by PKCδ (Murakami et al., 2002) which inhibits binding of PIP2 to syndecan-4, thus preventing PKCα activation. Syndecan-4-dependent translocation of PKCα to the membrane is critical for the assembly of mTOR complex 2 (mTORC2) and activation of AKT (Partovian et al., 2008).

Syndecan-4 has been reported to mediate canonical and non-canonical Wnt signaling pathways during embyogenesis. Escobedo et al. (2013) reported that syndecan-4 functions in the non-canonical Wnt signaling during embyogenesis in mice. This is evidenced by the fact that syndecan-4 knockout mice had defects in stereociliary bundle orientation in the mechanosensory hair cells in the inner ear (Escobedo et al., 2013), which resulted from the interrupted non-canonical Wnt signaling (Montcouquiol et al., 2006). Syndecan-4 is also involved in the canonical (Wnt/ β-catenin) pathway by regulating low-density-lipoprotein receptor-related protein, LRP6, and R-sponsin 3 (Astudillo et al., 2014).

Glypican-1 mediated cell signaling is largely through interacting with other proteins with its heparan sulfate chains (Figure 5). Glypican-1 heparan sulfate chains bind to FGF2 and are shed from the cell membrane through the cleavage action of phospholipases, thus sequestering FGF2 away from its FGFR receptors (Mythreye and Blobe, 2009; Velleman et al., 2013). This is supported by the report that cells deficient of glypican-1 have increased sensitivity to FGF2 (Gutierrez and Brandan, 2010).

In addition to FGF2 signaling, glypican-1 can regulate other signaling pathways, such as HGF, Wnt, transforming growth factor-beta, and hedgehog (Jackson et al., 1997; Shiau et al., 2010; Wilson and Stoeckli, 2013; Gutierrez et al., 2014). The mechanisms by which glypican-1 regulates cellular signaling pathways are fully understood.

## Heparan sulfate and N-glycosylation may have a role in the conversion of myogenic cells to an adipogenic lineage

Satellite cells are a multipotential mesenchymal stem cell population with plasticity to commit to myogenesis or alternative differentiation programs such as osteogenesis or adipogenesis (Asakura et al., 2001; Shefer et al., 2004). The first week posthatch in broilers has been shown to be the period of maximal satellite cell activity and satellite cells are sensitive to environmental stimuli including temperature and nutritional regime during this time (Halevy et al., 1998, 2000, 2001, 2006; Velleman et al., 2010, 2014a). Both hot temperatures and feed restrictions during the first week posthatch result in the conversion of satellite cells to an adipogenic lineage (Velleman et al., 2014b; Pietsun et al., 2017). Grassot et al. (2017) showed that increased levels of heparan sulfate and decreases in N-linked glycosylation promote preadipogenic differentiation in lieu of myogenic differentiation in isolated murine satellite cells. At this time, it is not known if satellite cell membrane associated proteoglycans like syndecan-4 and glypican-1 play a role in the conversion of satellite cells to an adipogenic cellular fate. However, the expression of glypican-1 has been shown to be altered by an immediate posthatch feed restriction. Velleman and Mozdziak (2005) harvested pectoralis major (breast) muscle tissue from pretreatment day 0 chicks and chicks either fed or feed deprived for 1, 2, or 3 days after hatch, and after day 3 feeding was resumed in the feed deprived birds until day 7. Glypican-1 expression was decreased in the muscle tissue from feed deprived birds at day 3 (*P* < 0.05), but by day 7 after reinitiating feeding on day 3, was significantly elevated compared to the muscle tissue for the chicks maintained on feed (*P* < 0.05). Chicks feed restricted during the first week after hatch have higher fat levels in the breast muscle at market weight (Velleman et al., 2014b). In addition, to the functions of syndecan-4 and glypican-1 in myogenic cell proliferation, differentiation, and FGF2 regulation, they may also promote adipogenic differentiation of satellite cells.

## Possible microrna regulation of syndecan-4 and glypican-1 during satellite cell proliferation and differentiation

MicroRNAs (miRNA) are small 20–25-nucleotide RNA sequences encoded in the genome, which post-transcriptionally regulate gene expression (Ameres and Zamore, 2013; Finnegan and Pasquinelli, 2013). “These small RNA sequences have been shown to regulate a number of aspects of skeletal muscle development including satellite cell senescence and expression of the satellite cell-specific marker Pax7 (Chen et al., 2010; Dey et al., 2011; Cheung et al., 2012; Koning et al., 2012; Harding et al., 2016).” Since satellite cells are a heterogeneous pluripotent population of stem cells, functional differences in satellite cell subpopulations may, in part, be due to miRNA regulation. MicroRNAs have been shown to affect satellite cell function including their proliferation and differentiation (Chen et al., 2006, 2010; Kim et al., 2006; Dey et al., 2011). Both syndecan-4 and glypican-1 have been shown to play essential roles in satellite cell proliferation, migration, and differentiation (Velleman et al., 2007; Shin et al., 2013). However, the mechanisms regulating syndecan-4 and glypican-1 gene expression have not been well-studied. Harding and Velleman (2016) identified 3 microRNAs, miR-128, miR-24, and miR-16, predicted to target syndecan-4 and glypican-1. Inhibitors of these microRNAs were transiently transfected into turkey breast muscle myogenic satellite cell cultures and proliferation and differentiation were measured. In general, inhibition of the miRNAs resulted in a general reduction in satellite cell proliferation and differentiation. MicroRNAs-128 and -24 also inhibit the migration of satellite cells to form differentiated myotubes (Velleman and Harding, 2017). The reports by Harding and Velleman (2016) and Velleman and Harding (2017) are the first demonstration of a role of these miRNAs in poultry myogenesis and suggest that miR-128, miR-24, and miR-16 may target genes integral for satellite cell proliferation and differentiation as other genes in addition to syndecan-4 and glypican-1 may be affected by these miRNAs. Since miRNAs imperfectly base pair to target genes, each miRNA can have multiple gene targets (Krek et al., 2005; Sanchez et al., 2013). Furthermore, miR-24 has been shown in myoblasts to negatively regulate transforming growth factor-β to promote differentiation (Sun et al., 2008). Transforming growth factor-β is a strong inhibitor of both myogenic cell proliferation and differentiation.

## Conclusions

The heparan sulfate proteoglycans, syndecan-4 and glypican-1 both play critical roles in the regulation of avian breast muscle satellite cell activity including but not limited to proliferation, differentiation, and migration. To regulate these cellular processes, syndecan-4 and glypican-1 must be dynamically expressed and function in a communication network transmitting signals especially from growth factors like FGF2 to the cell resulting in cell behavior changes. MicroRNAs may also function in regulating the gene expression of syndecan-4 and glypican-1 and contribute to their dynamic expression during myogenesis. Figure 5 summarizes the cellular pathways and growth factor interactions for syndecan-4 and glypican-1. In addition to modulating muscle development recent research suggests that heparan sulfate proteoglycans and their N-glycosylation chains could be important in the conversion of myogenic cells to an adipogenic lineage.

## Author contributions

All authors listed have made a substantial, direct and intellectual contribution to the work, and approved it for publication.

### Conflict of interest statement

The authors declare that the research was conducted in the absence of any commercial or financial relationships that could be construed as a potential conflict of interest. The reviewer LB declared a shared affiliation, though no other collaboration, with one of the authors SV to the handling Editor, who ensured that the process met the standards of a fair and objective review.
